# Positive Impact of UV Photography on Individual Sun Protection: A Swiss Feasibility Study

**DOI:** 10.3389/ijph.2024.1607604

**Published:** 2024-09-06

**Authors:** Tanguy Corre, Kathrine Zimmermann, Olivier Gaide, David Vernez, Jean-Luc Bulliard

**Affiliations:** ^1^ Unisanté, Center for Primary Care and Public Health and University of Lausanne, Lausanne, Switzerland; ^2^ Centre Hospitalier Universitaire Vaudois (CHUV), Lausanne, Switzerland

**Keywords:** personalized prevention, skin cancer, UV photo, feasibility study, Switzerland, skin aging, sun exposure, outdoor workers

## Abstract

**Objectives:**

This study evaluates the feasibility and impact of conveying personalized sun protection message supported by a UV photograph of the face in Switzerland.

**Methods:**

440 adults from 14 private and public sites associated with high sun exposure received a skin cancer prevention intervention composed of a facial UV-filtered photograph and individual counselling by a trained registered nurse. Pre-/post intervention surveys assessed sun protection of participants, their skin cancer risk and reasons for behavioural change.

**Results:**

The range of facial UV spots’ count per individual was very broad (0–590) and mainly determined by phototype, followed by age. Three months after the intervention, 61% of participants positively changed their sun protection habit both during leisure and at work. Use of all sun protection means increased. No factor could be specifically associated to that propension for change. The individualized message was perceived as the main motivation for change.

**Conclusion:**

Personalized sun protection messages supported by a facial UV photograph led to significant favourable behavioural change in a highly sun-exposed population of adults.

## Introduction

Excessive exposure to ultraviolet radiation (UVR) is the main causative factor for skin cancer, which is the most frequent cancer type in Caucasian populations [1, 2]. Despite long-held public health recommendations, sun protection interventions and screening campaigns [3–8], the incidence of both melanoma, the deadliest form of skin cancer, and keratinocyte skin cancer have increased for several decades, albeit with promising signs of attenuation in some populations where prevention has been sustained for many years [2, 9]. These trends have largely been attributed to widespread lifestyle changes towards positive perception of tanning and more intense UV exposure, and the concomitant rise in outdoor activities and holidays spent in high UV irradiance destinations [10, 11].

The modest impact on skin cancer incidence of past prevention messages and interventions has stressed the need of novel, more effective health promotion approaches. This is particularly relevant for subgroups such as adolescents and young adults, for whom future consequences of their current risk-prone attitude has little effect on changing their behaviour, and outdoor workers, for whom compliance with universal sun protection messages (seeking shade, avoiding peak irradiances (11:00–15:00), using sunscreen and wearing a hat, sunglasses and long sleeves) can be hindered by job-specific constraints and regulations [12–14]. Multi-component intervention programmes and appearance-focused sun prevention campaigns have evidenced promising results [12, 15–18]. UV photography intervention combined with photoaging information appear more effective than health-focused intervention in reducing UV exposure, as appearance concern is a driver for tanning attitude [19].

UV-filtered photographs capture the underlying skin photodamage that is invisible to the naked eye, making it easier for individuals to understand and visualize the long-term consequences of UV exposure. By revealing fine wrinkles (early sign of photoaging) and hidden white spots (guttate hypomelanosis, e.g., areas where protective pigment cells have been destroyed by UV radiation), UV photographs indicate what the skin may ultimately look like in the future without proactive measures to increase one’s sun protection. By incorporating UV photography into prevention campaigns, organizations and healthcare professionals can thus effectively sensitize people to the damage incurred by their current habits and encourage sun behavioural changes.

The primary aim of this study was to evaluate the feasibility and the impact of a multicomponent, personalized skin cancer prevention intervention based on a facial UV photograph accompanied by an individual prevention message. Based on current evidence, it assumes that the effectiveness of such an intervention was established [19]. Unlike many earlier studies that focused on teenagers and populations of British descent, this study predominantly targets highly sun-exposed adults in Switzerland, a country that experiences a high incidence of melanoma and non-melanoma skin cancer in Europe [20–22] despite longstanding primary and secondary prevention campaigns [3, 5, 23]. The secondary aims of the study were to investigate 1) individual factors influencing skin damage due to UV exposure, and 2) drivers of the propension to change after being confronted to his/her own UV photograph.

## Methods

### Study Population

The study area covered the canton of Vaud in the western, French-speaking part of Switzerland. Outdoor workers and people with recreational activities associated with high exposure to solar UVR were the target group of the study. Medium to large size enterprises employing year-long outdoor workers, vocational schools training future outdoor professionals, as well as private and public entities in relation with outdoor leisure (garden centres, sport associations, swimming pools, organizers of outdoor events) were identified within the study area. From 45 entities contacted, 11 outdoor work businesses, 2 vocational schools and 1 swimming pool responded and participated.

The project was conducted in adherence with the Swiss Association of Research Ethics Committees, after receiving ethical approval, and with the 1964 Helsinki declaration and its later amendments or comparable ethical standards. The study protocol specified the legal characteristics of the study (age above 18 for participation, data management, study information material, personal rights, and confidentiality). Participation was on a voluntary basis and informed consent was required prior to data collection. Data were anonymized and irreversibly de-identified before analysis.

### Data Collection

The intervention was scheduled with and provided at the sites of the various institutions, businesses and events via the Unisanté health bus to facilitate participation during working hours. It took place during 8 weeks from May to August 2022. Participants presented themselves according to a specific individual appointment system. They filled a baseline questionnaire before having a UV picture taken of their face and receiving individual oral counselling by specially trained staff. Four registered nurses were trained about skin cancer and its prevention by a senior dermatologist (O.G.) in a 1-day on-site training with graphical and illustration content and dedicated time for theoretical and practical questions. The registered nurses also ensured that the questionnaire was entirely filled and answered any comprehension issue participants may have with it. The electronic questionnaire developed with REDCap [24, 25] covered socio-demographic data, phototype, history of melanoma, and information about sun exposure (see Table 1; Supplementary Table 1). Phototype was assessed by two means: the participant’s self-determined phototype according to provided descriptions [26] and the phototype as interpreted by the camera software.

**TABLE 1:** Population characteristics (adults with occupational or high recreational sun exposing activity, canton of Vaud, Switzerland, 2022).

Study variable (unit)	Baseline sample (n = 440)	Non-respondents to follow-up (n = 166)	Respondents to follow-up (n = 274)	*p*-value*
Age (mean, in years)	42.2	39.5	43.8	<0.05
Gender (female)	42%	37%	45%	NS
Education level attained				<0.001
Mandatory school	8%	14%	4%	
Apprenticeship	42%	46%	39%	
High school diploma	5%	4%	6%	
Advanced professional education	12%	10%	13%	
University or higher institute of applied science	33%	25%	38%	
Melanoma history				NS
Yes, personal	1%	1%	1%	
Yes, familial	8%	4%	9%	
No	91%	94%	89%	
Outdoor worker	52%	54%	50%	NS
Exposure to sun during leisure	95%	95%	94%	NS
Outdoor sport practice	70%	69%	70%	NS
Sunbathing practice	40%	45%	38%	NS
Holidays at sunny places (per year)				NS
Yes, more than 2 weeks	54%	52%	55%	
Yes, less than 2 weeks	42%	42%	41%	
None	5%	6%	4%	
Sunbed use	14%	8%	18%	<0.05
Sunburn during childhood				NS
Yes	60%	61%	60%	
No	35%	37%	33%	
No recall	5%	2%	7%	
Global score of sun protection (mean)	2.4	2.1	2.5	<0.001
Use of natural shadow	60%	54%	63%	NS
Wearing long sleeves	7%	4%	9%	NS
Wearing hat	52%	47%	54%	NS
Wearing sunglasses	68%	61%	72%	<0.05
Use of sunscreen	50%	40%	55%	<0.001
Skin phototype				<0.05
I	6%	8%	5%	
II	37%	31%	41%	
III	48%	49%	47%	
IV-V-VI	9%	13%	7%	
Number of wrinkles (mean)	65.5	64.2	66.3	NS
Number of UV spots (mean)	343.1	307.5	364.6	<0.001

Due to rounding, the sum of categories for variables may not exactly add up to 100%.

* *p*-value of the test assessing the significance of the difference between respondents and non-respondents (t-test for continuous variables, chi-square test for categorical variables).

NS, Not Significant.

A non-medical UV camera device (VISIA 7 from Canfield^®^) was used to take the picture and count the UV spots (white spots invisible to the human eye, reflecting past UV exposure) and fine wrinkles on the face. A follow-up questionnaire was sent at least 3 months after participation, by post or email (Supplementary Table 2). A brief survey was sent to all participating entities to evaluate their overall satisfaction with the intervention.

### Sun Protection Scores

A global sun protection score was built from five protection measures assessed: use of shade, wear of long sleeves, hat, sunglasses, and application of sunscreen. One point per measure used was given leading to a theoretical maximum sun protection score of five. A more detailed score of frequency of sun protection use was computed by attributing from zero to three points per protection item according to the reported frequency of use (0 = never, 1 = sometimes, 2 = often, 3 = always), generating a score on a 0 to 15 points scale.

The measure of behavioural change in sun protection was assessed through five questions in the follow-up questionnaire (Supplementary Table 2). Each question addressed any change in a specific sun protection item assessed at baseline. For each item, participants could indicate whether they used it since the intervention more (+1), as much (0) or less (−1) than at baseline. It resulted in a global sun protection change score. This assessed score could differ from the perceived sun protection change score which was directly derived from a question prompting participants whether they felt having globally changed their sun protection after the intervention.

### Statistical Analyses

All statistical analyses were performed using the R software version 4.2.1. Correlations were assessed using the Pearson correlation coefficient. Differences between respondents and non-respondents to the follow-up questionnaire were assessed by t-test for continuous variables and chi-square test for categorical variables. Bivariate association analyses were performed using simple linear regressions. Multivariate multi-level analyses were performed by linear mixed models using the collection site as random effect, then subjected to a backward regression procedure following the Akaike information criterion. A likelihood ratio test comparing the models with and without that random effect was performed to assess the statistical significance of the random effect. An alpha level of 0.05 was set for statistical tests.

## Results

### Study Population

A total of 440 subjects were recruited. Population’s characteristics are summarized in Table 1. Mean age was 42 years old (median = 44; SD = 13.8), with 42% of women. Some 33% of subjects had tertiary education and 9% reported a personal or familial history of melanoma. About half of the recruited population was composed of outdoor workers (52%). Most people (95%) experienced recreational sun exposure, with over half spending over 2 weeks a year of holidays in high UV irradiance areas (54%), and 14% reported to have used sunbed in their life.

The most frequently reported sun protection measures were wearing sunglasses (68%), using natural shadow (60%), followed by wearing a hat (52%) and applying sunscreen (50%). Wearing long sleeves as a sun protection means was uncommon (7%).

Correlation between the phototype automatically interpreted by the UV camera software and the self-assessed phototype was moderate (*R* ^2^ = 0.48) with no phototype I and VI detected by the software. We thus used the self-assessed phototype for our analysis as it is the recommended method in dermatology [26, 27].

The average number of UV spots per participant was 343 (median = 366; SD = 140), ranging from 0 to 590 in a pseudo normal distribution, left tailed (Supplementary Figure 1). The average number of wrinkles, also computed by the camera software, was 65 (median = 62; SD = 35) per participant. Counts of UV spots and wrinkles were weakly correlated (*R* ^2^ = 0.16).

62% of participants (n = 274) responded to the follow-up questionnaire. Differences between respondents and non-respondents are shown in Table 1. The most significant differences (*p* < 0.001) were that respondents had more UV spots (+57.1), a higher global sun protection score (+0.4), and attained a higher level of education (38% vs. 25% of tertiary education). Other significant differences were a greater age (+4.3 years), greater use of sunbed (18% vs. 8%), a more sun-sensitive phototype (46% vs. 39% of phototype I or II) and a higher use of all sun protection means, particularly sunscreen (55% vs. 40%), among respondents than non-respondents.

### Determinants of UV Spots’ Count

Bivariate analyses indicated that age, educational level, phototype, wrinkles’ count, sunbathing practice, use of sunbed, sunburns during childhood and both sun protection scores were significantly associated (*p* < 0.001) with the number of UV spots (Supplementary Table 3). Phototype showed the highest explained variance (*R* ^2^ = 0.25, *p* < 0.001): the more sensitive the skin type, the higher the number of UV spots. People of phototype I had in average 251 more UV spots on their face than individuals with phototype IV, V or VI. Both the type of collection site (company vs. leisure vs. school) and the collection site itself were also strongly associated to the number of UV spots observed (*p* < 0.001).

Results from the multivariate analysis are shown in Table 2. Age, gender, education, phototype, sunbed use and wearing long sleeves were retained as factors in the model explaining best the number of observed UV spots, but not the collection site (as random effect in the model, *p* = 0.452). Phototype and age were the most significant factors (*p* < 0.001). Each additional year of age increased the average number of UV spots by 4.9. The gradient observed in the bivariate analysis for the phototype persisted: each category of phototype from IV-VI to I showed a greater number of UV spots, with a maximal amplitude of 213 more UV stains for group I phototype members compared to the least sun-sensitive phototype group. Women had in average 21 more UV spots than men (*p* < 0.05). Education, wearing long sleeves and using sunbeds contributed to increase the variance of the model without being individually significantly associated with the number of UV spots.

**TABLE 2:** Multivariate analysis of determinants of UV spots count (adults with occupational or high recreational sun exposing activity, canton of Vaud, Switzerland, 2022).

Variable	Coefficient	CI 95%
Age, per year	4.9	4.2; 5.6
Female gender	20.9	1.3; 40.4
Skin phototype (ref: IV-V-VI)		
I	212.6	164.5; 260.7
II	190.4	156.4; 224.5
III	103.3	70.5; 136.2
Highest education (ref: mandatory school)		
Apprenticeship	−27.9	−64.5; 8.7
High school diploma	−3.4	−55.1; 48.2
Advanced professional education	6.3	−36.1; 48.7
University or higher institute of applied science	−35.6	−73.4; 2.2
Sunbed use	20.8	−6.9; 48.5
Wearing long sleeves	28.5	−7.3; 64.4

The adjusted *R* ^2^ of the final model was 0.53.

### Post-Intervention Change in Sun Protection

**FIGURE 1:**
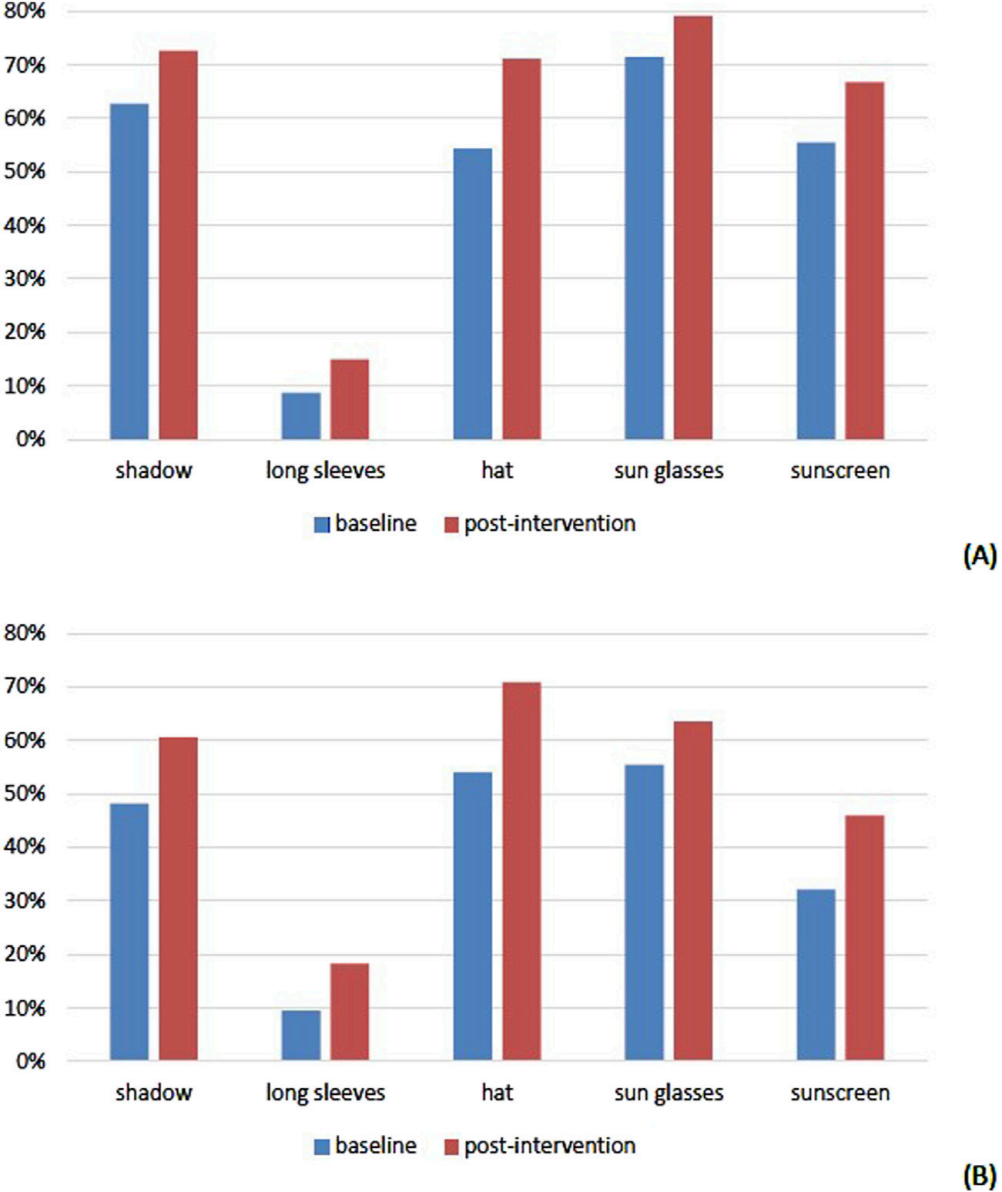
Percentage of users for each sun protective measure during leisure [**(A)**, n = 274] and at work [**(B)**, n = 137]: comparison between baseline and post-intervention (adults with occupational or high recreational sun exposing activity, canton of Vaud, Switzerland, 2022).

Comparison of use of sun protection measures at baseline and post-intervention showed statistically significantly higher use of each sun protection measure at individual level during leisure (Figure 1A) and, for outdoor workers, at work (Figure 1B). During leisure, the highest absolute increase was observed for wearing a hat (from 54% before to 71% after the intervention), and the highest relative increase was observed for wearing long sleeves, which was the least used protection overall, with a 1.7-fold increase (from 9% to 15%). Results were similar at work with the same highest absolute increase observed for wearing a hat, and a 2-fold increase for wearing long sleeves (9%–18%). Globally, sun protection was more frequently used during leisure than at work, sunscreen being the protection harbouring the highest difference between the two settings (at baseline: 55% vs. 32%, after intervention: 67% vs. 46%).

**FIGURE 2:**
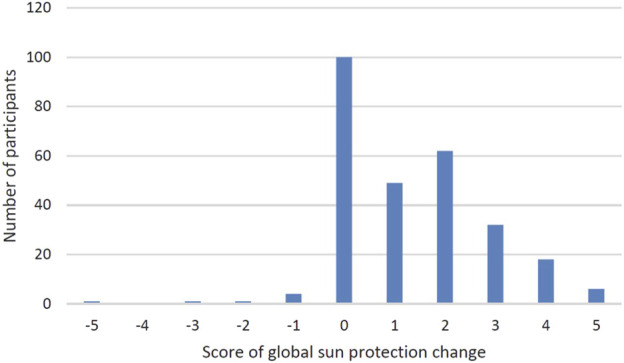
Distribution of score of global sun protection change during leisure (adults with occupational or high recreational sun exposing activity, canton of Vaud, Switzerland, 2022).

The distribution of the global sun protection change score (as defined in the methods) is shown in Figure 2. Only seven participants reported a global decrease in their protection (negative score). A null score, indicating no global change, was found for 100 participants (36% of respondents). Most participants (n = 167, 61%) increased globally their protection, mainly moderately (scores of +1 or +2). Association analyses of determinants of the global sun protection change score could not identify any significant factor, and the multivariate analysis only explained 6% of the variance (data not shown). Outdoor workers were asked specifically to assess their change towards sun protection at work. The distribution of the global sun protection score change at work was very similar to that observed for leisure in Figure 2 (Supplementary Figure 2).

Participants who reported a perceived global change in their sun protection behaviour were prompted about their motivations (n = 135). Most pointed at the individualized message delivered by the registered nurse, rather than the UV photograph, as being the main reason for having changed their sun protection (n = 84, 62%, data not shown). Knowledge of one’s own number of UV spots did not seem to directly influence motivation. As a reason for not having changed or not enough one’s sun protection habit (n = 152), most respondents evoked their good practice at baseline and thus a recommendation from the nurse to pursue in this way (n = 104). Others mentioned reasons were the non-feasibility at work (n = 29) and the constraining character of the protection measures (n = 15). Only four participants disagreed with or did not understand the recommendation.

## Discussion

This intervention, which exposed 440 volunteers to their facial UV-filtered photograph and a personalized sun protection counselling by a trained registered nurse, evidenced favourable behavioural change in sun protection measures in the following months. These changes were observed in 6 out of 10 participants, during leisure and occupational activities alike, and appeared to be homogeneous across all sociodemographic and skin cancer risk factors measured.

Overall, our results support the positive motivational effect and impact on sun-related behaviour of skin cancer prevention intervention based on appearance [28, 29]. Use of all sun protection measures significantly improved. The increased proportion of people who reported after the intervention staying out of the sun in the middle of the day, the most impactful protection measure, is particularly promising. Comparison with other studies is however challenging due to the heterogeneity of interventions, and large differences in target populations and outcomes.

Previous interventions were often targeted at children and teenagers and conducted predominantly in US and Australian populations [16, 19, 30, 31]. A recent cluster-randomized trial among Brazilian pupils promisingly showed that a face-aging mobile app in which an image is altered to predict future appearance improved skin cancer protection behaviour meaningfully but less tanning behaviour [32]. Better sun protection may not translate into reduced sun exposure, as motivating factors for sun protection behaviour can differ from those for sun exposure. In a US multi-component intervention including UV photography, an increase in sun protection but not in sun exposure was observed in the intervention group [33]. In another intervention based on UV photography and photoaging information, with objective assessment of change in skin colour by spectrophotometry measurement, there was clear evidence of less skin darkening and better sun protection behaviour 1 year after intervention among predominantly non-Caucasian undergraduate US students [30].

Our finding of an overall improvement in sun protection with little to no effect on specific behaviours that have already been vigorously promoted is in line with a sun protection intervention in Australia [31]. Earlier studies have reported that Swiss people largely know how to protect themselves from the sun [11, 12, 34, 35]. Self-induced improvement in one’s protection may likely start with one’s most convenient means. This concurs with our observation of the largest favourable change in hat wear, which can also be driven by fashion or discomfort from direct sunlight in the face. Differences observed in use of sun protection means during leisure and work and their potential improvement are also likely related to occupational constraints. For instance, self-reported sun protection of outdoor workers was substantially higher in our study than in a series of agricultural workers in Switzerland [12].

The intervention was positively perceived by both participants and enterprises. Retention rate was in the range of previous studies, albeit prior interventions often provided incentives to participants in order to reduce attrition [30].

Our study results evidenced the wide disparity in number of facial UV spots between individuals, with phototype as clearly the main predictor of this number. Darkest skin revealed far lesser spots, from none detected in the extreme case, to several hundreds for the lightest skin complexion. Each year of age led to the detection, on average, of five additional facial areas where protective pigment cells had been destroyed. The greater number of UV spots found for women, after controlling for other measured factors might be related to the difficulty of the UV photography to identify unpigmented areas when covered by hair such as beard or moustache.

Although the main identified determinants (age, sex and phototype) of the number of UV spots on someone’s skin cannot be modified by prevention, it informs on one’s photo-susceptibility and may prompt behavioural changes to prevent further cell damage. Our finding that people with lighter skin type were, despite their overall higher sun protection, more prone to higher UV spots counts - hence at higher risk of skin cancer - confirmed evidence that, when sun exposed, the intrinsic higher risk of susceptible subjects often cannot be compensated by their better than average degree of sun protection [11, 34, 36]. To the best of our knowledge, this is the first study presenting population-level data on the distribution of total number of facial UV spots. Our series provide comparative values for other predominantly highly sun-exposed Caucasian populations with similar sociodemographic features, host and risk factors. However, comparability across camera devices in the identification and counting of UV spots (i.e., counting one large spot vs. several spots within a cluster of small spots, detection of UV spots in presence of facial hair) might warrant investigation.

Our feasibility study has limitations. Data were mainly self-reported with a selectively higher compliance in the post intervention of more sun-sensitive participants, which might have led to a greater impact of the intervention. Albeit internal data cross-validation and comparisons with other Swiss data did not reveal any large or unexplained inconsistency, some desirability bias cannot be discarded. The study population was slightly better educated and had a marginally more sun-sensitive skin than the general Swiss population (45% vs. 36% with a tertiary education and 91% vs. 86% with a phototype I, II or III). However, representativity was not a study aim, and the self-selection of higher risk people corresponded to the intended target group for such an intervention. We also acknowledge that an objective assessment of change in sun protection practice was beyond the scope of our study. There was, however, no reasonable ground to assume a differential bias in self-reported practice between baseline and post-intervention.

The study design without control group did not allow to draw a conclusion on the impact of the UV photography alone versus the whole intervention with the adjunct personalized counselling. Albeit the main self-perceived reason for change was the individualized message, individual protection recommendations were often similar in absence of evidence of a high UV exposure on the UV photograph. Consequently, these results cannot rule out the role of the UV picture, independently from the number of UV spots detected.

Our study also has several strengths. Our outcome was based on recent sun protection behaviour rather than intended protection, which might not eventuate and be less accurate. Our study was sizeably larger than most earlier ones [28], investigated potential drivers of sun behavioural change which have little been explored so far [32], and is the first intervention of this kind in an European adult population. Unlike many prior studies with follow-up limited up to 1 month [28], which might be short to observe behavioural changes, we followed-up participants at 3 months during the crucial summertime period. Longer follow-up up to 1–2 years may reduce compliance, expose to the influence of factors unrelated to the intervention, and include seasons where sun protection is less or not recommended.

Overall, the intervention had a significant positive impact on individual sun protection, especially through wearing more covering clothes (long sleeves and hats), regardless of the number of UV spots revealed by the UV picture. Such intervention’s set up could be further utilized as a public health measure to increase population’s sun protection. Further studies would be needed to establish the actual role of the UV photography in that observed change. Potential mechanisms that led our intervention to the observed changes might include a cue to action, increased awareness, reminder or compliance with sun protection measures, or fear. Another question left open by the design of this study concerns the perennity over long periods (several years or even decades) of the change observed in our design three to 6 months after the intervention. While a previous study seemed to indicate that the effect lasts for 12 months [30], another follow-up several years later could help answering this question.
