# Supplementary material for: Positive Impact of UV Photography on Individual Sun Protection: A Swiss Feasibility Study
Source: Int J Public Health. 2024 Sep 6;69:1607604. doi: 10.3389/ijph.2024.1607604 (PMC11412796; doi:10.3389/ijph.2024.1607604)
Supplement: Supplementary file 1 [file Table1.pdf]

Supplementary material

Supplementary Tables and Figures captions

Supplementary Figure 1: Distribution of UV spots count in the study population (adults with occupational or high recreational sun exposing activity, canton of Vaud, Switzerland, 2022)

Supplementary Figure 2: Distribution of score of global sun protection change at work (adults with occupational sun exposure, canton of Vaud, Switzerland, 2022)

Supplementary Table 1: Baseline questionnaire (adults with occupational or high recreational sun exposing activity, canton of Vaud, Switzerland, 2022)

Supplementary Table 2: Post-intervention questionnaire (adults with occupational or high recreational sun exposing activity, canton of Vaud, Switzerland, 2022)

Supplementary Table 3: bivariate analysis of UV spots count by study variable (adults with occupational or high recreational sun exposing activity, canton of Vaud, Switzerland, 2022)

Supplementary Figure 1

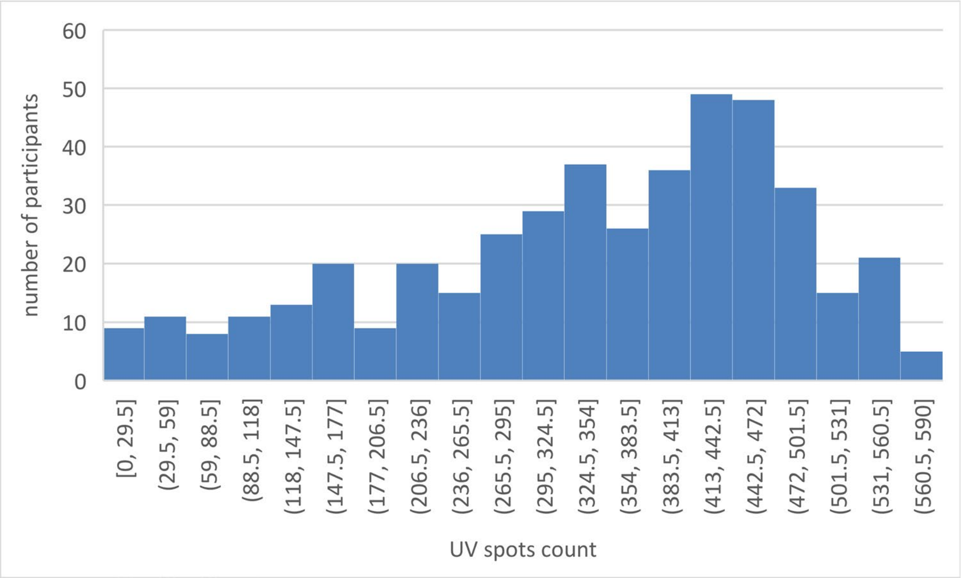

Supplementary Figure 2

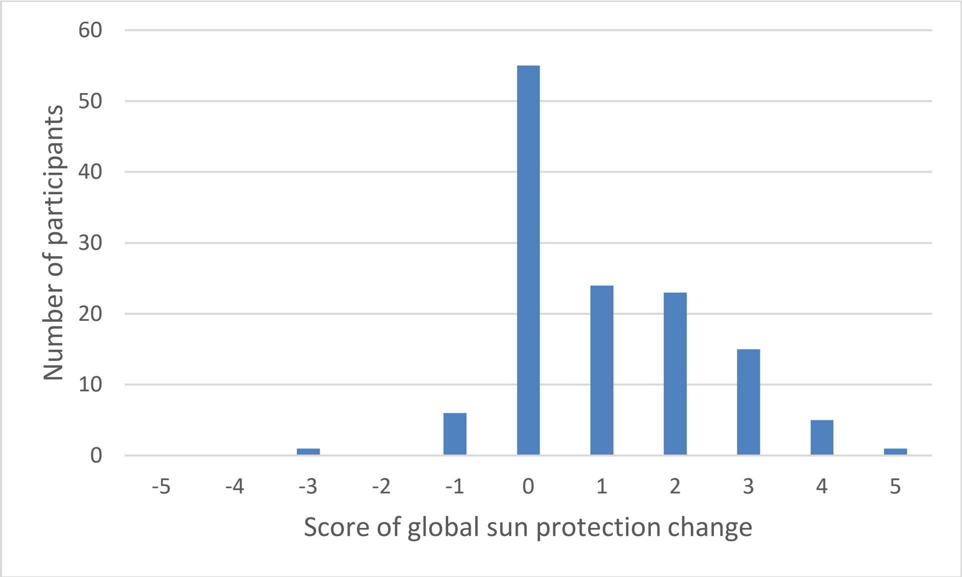

Supplementary Table 1

0- How would you like to receive the follow-up questionnaire in a few weeks?
Email : ____________ / Postal address: _______________

1- Gender
Male / Female / Other

2- Year of birth : YYYY

3- What is your highest level of education?
Compulsory school (up to age 16) / Secondary level II: vocational training / Secondary level II: general education / Tertiary level I: higher vocational training / Tertiary level II: college or university

4- Do you work or have you worked outdoors? No / Yes
If yes, for how many years? 1 or less / more than 1, up to 5 / more than 5, up to 10 / more than 10

5- Do you expose yourself to the sun outside of work? No / Yes
If yes: When doing sport / sunbathing / Other
If yes, for how many years? 1 or less / more than 1, up to 5 / more than 5, up to 10 / more than 10

6- Do you have a personal or family history of melanoma (parents, children, brother, sister)?
No / Yes, personal / Yes, family / Don't know / Don't wish to answer

7- Skin type
Very fair skin, tanning: never or very light, sunburn: always severe / Fair skin, tanning: barely, sunburn: generally severe / Slightly matt skin, tan: good with gradual exposure, sunburn: sometimes moderate / Very matt skin, tanning: rapid, sunburn: rare / Dark skin, sunburn: very rare / Black skin, sunburn: almost never

8- If you work outdoors (otherwise, go on to the next question):
At work, to protect yourself from the sun, do you use :
Shade, natural or structural (building, canvas, etc.)? Never / Sometimes / Often / Always
Long-sleeved clothing? Never / Sometimes / Often / Always
A hat (cap, scarf etc.)? Never / Sometimes / Often / Always
Sunglasses? Never / Sometimes / Often / Always
Sunscreen? Never / Sometimes / Often / Always

9- In your spare time, to protect yourself from the sun, do you use :
Shade, natural or structural (building, canvas, etc.)? Never / Sometimes / Often / Always
Long-sleeved clothing? Never / Sometimes / Often / Always
A hat (cap, scarf etc.)? Never / Sometimes / Often / Always
Sunglasses? Never / Sometimes / Often / Always
Sunscreen? Never / Sometimes / Often / Always

10- When you are exposed to the sun, whether for work or leisure, do you do so between 11am and 3pm? Yes, absolutely / Rather yes / Rather no / Never

11- Sun exposure in adulthood (from age 18)
Number of holiday weeks per year with intense sun exposure ? 0 week / Up to 2 weeks / more than 2 weeks
Do you currently use sunbed or have you done so in the past? No / Yes, up to 20 times a year / Yes, 21 times a year or more
For how many years have you used sunbed? ___ year(s)

12- Did you suffer from severe sunburn before the age of 18? (painful redness or blisters lasting at least 2 days) No / Yes / I don't remember

POST-PHOTO QUESTION
13- Do you think the photo will encourage you to follow the recommendations we will be giving you in a moment? Yes absolutely / Rather yes / No / I don't know

Supplementary Table 2

1- Identifier

2- Year of birth : YYYY

3- Since the UV photo was taken and the recommendation made, do you feel that you have increased your level of protection against the sun? Yes, I have increased my protection / No, I have not changed my protection /No, I have reduced my protection

4- Details of your changes in sun protection behaviour
If you work outside, as part of your job, did you use
Natural or structural shading (building, canvas, etc.)? Less than before / As much as before / More than before
Long-sleeved clothing? Less than before / As much as before / More than before
A hat? Less than before / As much as before / More than before
Sunglasses? Less than before / As much as before / More than before
Sunscreen? Less than before / As much as before / More than before
During your free time, when exposed to the sun, did you use
Natural or structural shading (building, canvas, etc.)? Less than before / As much as before / More than before
Long-sleeved clothing? Less than before / As much as before / More than before
A hat? Less than before / As much as before / More than before
Sunglasses? Less than before / As much as before / More than before
Sunscreen? Less than before / As much as before / More than before

5- Have you reduced your exposure to the sun between 11am and 3pm?
Yes, I was less exposed between 11am and 3pm / No, I was just as exposed or more exposed between 11 and 3pm / Not applicable (I was not exposed to the sun)

6- Have you reduced your visits to sunbeds ? Yes / No / Not applicable (I was not using them)

7- [if yes to question 3] Why did you change your behaviour?
The message was clear / The photo was frightening / The message was clear and the photo frightening
[If no to question 3] Why didn't you change your sun exposure behaviour? (Several answers possible)
The recommendation indicated that my behaviour was appropriate / I found the recommendation too restrictive / I did not understand the recommendation / I did not agree with the recommendation / I found the recommendation not feasible at work

8- Was the recommendation easy to understand? Yes, completely / Rather yes / Rather no / Not at all

9- Was the UV photo-taking procedure tedious? Not at all / Not really / Yes, a little / Yes, a lot

10- Did you find the UV photo revealing, that it gave you relevant information that you didn't know?
Yes, completely / Rather yes / Rather no / Not at all

11 Would you have been convinced by the recommendation in the absence of a photo?
Yes, completely / Rather yes / Rather no / Not at all

12- Before taking part in this project, did you think you had a similar level of risk to that identified during the intervention? Yes / No

13- Would you have been prepared to travel to have your UV photo taken (service not provided at work or leisure centre, etc.)? Yes / No

If yes - Would you have been prepared to pay all or part of the cost of the service [indicative budget= 50 Swiss francs]? Yes, in full / Yes, in part / No

14- Are you satisfied with the service? Yes, completely / Rather yes / Rather no / Not at all

Supplementary Table 3

Study variable (reference category)	Coefficient	p-value
Age, per year	5,7	<0.001
Female gender	-13,4	NS
Education level attained (ref: apprenticeship)		<0.001
mandatory school	47.9	NS
high school diploma	37.4	NS
advanced professional education	87.7	<0.001
university or higher institute of applied science	-7.9	NS
Melanoma history (ref: no)		NS
yes, personal	12.1	NS
yes, familial	15.7	NS
Outdoor worker (ref=no)	-13.9	NS
Exposure to sun during leisure (ref=no)	-22.1	NS
Outdoor sport practice (ref=no)	-22.2	NS
Sunbathing practice (ref=no)	-44.6	<0.001
Holidays at sunny places (per year) (ref: none)		<0.05
yes, more than two weeks	-65.1	<0.05
yes, less than two weeks	-29.5	NS
Sunbed use (ref=no)	73	<0.001
Sunburn during childhood (ref : yes)		<0.001
no	-63.8	<0.001
no recall	-7.7	NS
Global score of sun protection, per point	22.9	<0.001
Score of frequency of sun protection, per point	11.9	<0.001
Use of natural shadow (ref=no)	23.6	NS
Wearing long sleeves (ref=no)	75.3	<0.05
Wearing hat (ref=no)	41.0	<0.05
Wearing sunglasses (ref=no)	25.6	NS
Use of sunscreen (ref=no)	42.7	<0.05
Skin phototype (ref : IV+V+VI)		<0.001
I	250.7	<0.001
II	222.0	<0.001
III	118.0	<0.001
Number of wrinkles	0.6	<0.001
